# Antimitotic activity of DY131 and the estrogen-related receptor beta 2 (ERRβ2) splice variant in breast cancer

**DOI:** 10.18632/oncotarget.9719

**Published:** 2016-05-30

**Authors:** Mary M. Heckler, Tizita Zewde Zeleke, Shailaja D. Divekar, Aileen I. Fernandez, Deanna M. Tiek, Jordan Woodrick, Alexander Farzanegan, Rabindra Roy, Aykut Üren, Susette C. Mueller, Rebecca B. Riggins

**Affiliations:** ^1^ Department of Oncology, Lombardi Comprehensive Cancer Center, Georgetown University Medical Center, Washington, DC 20057, USA

**Keywords:** ESRRB, ERRbeta, cell death, mitosis, p38 MAPK

## Abstract

Breast cancer remains a leading cause of cancer-related death in women, and triple negative breast cancer (TNBC) lacks clinically actionable therapeutic targets. Death in mitosis is a tumor suppressive mechanism that occurs in cancer cells experiencing a defective M phase. The orphan estrogen-related receptor beta (ERRβ) is a key reprogramming factor in murine embryonic and induced pluripotent stem cells. In primates, ERRβ is alternatively spliced to produce several receptor isoforms. In cellular models of glioblastoma, short form (ERRβsf) and beta2 (ERRβ2) splice variants differentially regulate cell cycle progression in response to the synthetic agonist DY131, with ERRβ2 driving arrest in G2/M.

The goals of the present study are to determine the cellular function(s) of ligand-activated ERRβ splice variants in breast cancer and evaluate the potential of DY131 to serve as an antimitotic agent, particularly in TNBC. DY131 inhibits growth in a diverse panel of breast cancer cell lines, causing cell death that involves the p38 stress kinase pathway and a bimodal cell cycle arrest. ERRβ2 facilitates the block in G2/M, and DY131 delays progression from prophase to anaphase. Finally, ERRβ2 localizes to centrosomes and DY131 causes mitotic spindle defects. Targeting ERRβ2 may therefore be a promising therapeutic strategy in breast cancer.

## INTRODUCTION

Breast cancer remains a leading cause of cancer-related death in women [1, 2]. The prognosis for patients with hormone receptor-positive and/or human epidermal growth factor receptor 2 (HER2)-positive disease has been significantly improved by anti-estrogen and/or anti-HER2 therapies. By contrast, triple negative breast cancer (TNBC), which lacks hormone receptors and HER2, accounts for 13% of all breast cancers. TNBC is most prevalent in racial and ethnic minority women [3] and lacks clinically actionable therapeutic targets [4].

Mitotic catastrophe, or death in mitosis (DiM), is a tumor suppressive mechanism that occurs in cancer cells experiencing a defective M phase, ultimately leading to apoptosis or other forms of cell death [5, 6]. DiM is associated with a number of established cytotoxic chemotherapies and emerging targeted agents such as microtubule targeting drugs (vinca alkaloids and taxanes), inhibitors of mitotic entry and checkpoint kinases, and inhibitors of the multi-protein anaphase promoting complex/cyclosome (APC/C) or the spindle assembly checkpoint (SAC) [7]. However, there are significant liabilities inherent to some of these existing approaches, particularly those targeting microtubules because they are essential for cytoskeletal structure and vesicle trafficking outside of mitosis. An incomplete mitotic block allows slippage into the next G1 phase where, in the presence of apoptotic defects that may prevent elimination of these cells, chromosomal instability can lead to more aggressive tumor behavior. Other approaches include the inhibition of centrosome clustering, a mechanism to complete bipolar division used by cancer cells with centrosomal amplification; small molecules which inhibit this process are being developed as novel antimitotic therapies in breast and other cancers [8–11]. MYC-mediated modulation of pro-apoptotic BH3-only proteins has also recently been implicated in the control of DiM [12].

An attractive approach to inducing DiM that may avoid some pitfalls of microtubule inhibitors could be to inhibit a target protein that is enriched in cancer vs. normal cells and has pro-tumorigenic roles in both mitosis and interphase (e.g. survivin/BIRC5) [7]. Activation of a tumor suppressor pathway with mitotic and non-mitotic functions would be similarly beneficial. Nuclear receptors have historically been very successful targets for drug development. Estrogen-related receptors (ERRs) are orphan members of this protein family that have no known endogenous ligands, although their function can be modified by synthetic ligands [13–15] and the abundance of binding partners [16–19]. In mice, ERRβ (ESRRB/ERR2/ERRbeta/ESRL2/NR3B2) has emerged as a key reprogramming factor in embryonic and induced pluripotent stem cells [20–22], where it promotes a more permissive G1/S checkpoint [23, 24]. By contrast, exogenous expression of human ERRβ has tumor suppressive activities that activate the G1/S checkpoint through the induction of CDKN1A (p21) in prostate cancer cells [25]. The discrepancy in mouse vs. human function of ERRβ may be explained, in part, by alternative splicing [26]. Lower organisms express only the short form of ERRβ, a transcriptionally active nuclear receptor of 433 amino acids hereafter referred to as ERRβsf. In primates, however, the ESRRB locus contains three additional exons and gives rise to two other known transcripts with alternative carboxyl-terminal extensions: ERRbeta2 (ERRβ2, 500 amino acids) and ERRβ-Δ10 (508 amino acids) [26]. We have recently demonstrated that in cellular models of glioblastoma (GBM), the ERRβsf and ERRβ2 splice variants differentially regulate cell cycle progression in response to a synthetic ERR agonist, DY131 [27, 28]. As in prostate cancer [25], DY131-stimulated ERRβsf mediates a G1 arrest concurrent with the induction of p21. However, activation of ERRβ2 by this ligand drives arrest in G2/M [27].

The goals of the present study are to determine the cellular function(s) of ligand-activated ERRβ splice variants in breast cancer and evaluate the potential of DY131 to serve as an antimitotic agent, particularly in TNBC. DY131 inhibits growth in a diverse panel of breast cancer cell lines, causing cell death that involves the p38 stress kinase pathway and a bimodal cell cycle arrest. ERRβ2 facilitates the block in G2/M, and DY131 delays progression from prophase to anaphase. Finally, ERRβ2 is a cytosolic protein that also localizes to centrosomes, and DY131 treatment leads to the appearance of multi- and monopolar spindles. Activation of ERRβ, particularly the ERRβ2 splice variant, may therefore be a promising therapeutic strategy in breast cancer.

## RESULTS

### ERRβ2 has no transcription factor activity in breast cancer cells

**Figure 1:**
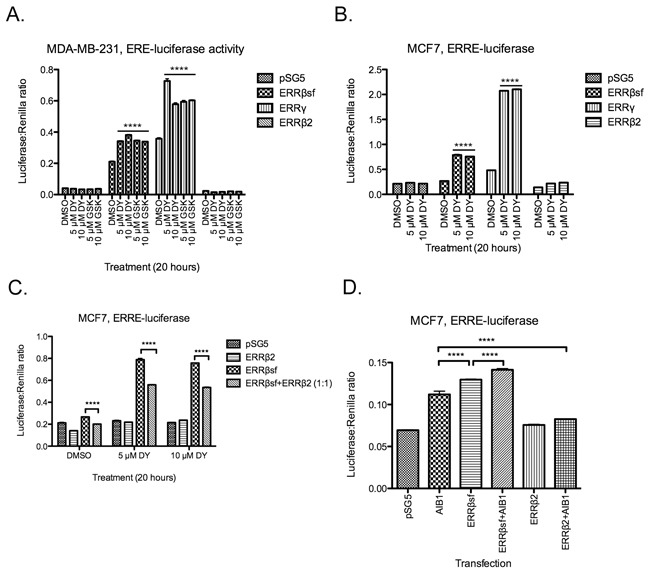
ERRβ2 has no transcription factor activity in breast cancer cells MDA-MB-231 **A.** and MCF7 cells **B.** transiently co-transfected with the indicated promoter-reporter luciferase constructs and receptor cDNA, then treated with either DY131 (DY), GSK4716 (GSK), or DMSO control (18-20 h) as shown. N = 3 for a representative assay peformed in triplicate, two-way ANOVA with Bonferroni post-test. **** denote post-hoc Bonferroni comparison for each drug treatment vs. DMSO control. C, MCF7 cells transiently co-transfected with ERRE-luciferase and ERRβ2, ERRβsf, or a 1:1 ratio of the two cDNAs, then treated with DY131 or DMSO control for 18-20 h. N = 3 for a representative assay performed in triplicate, two-way ANOVA with Bonferroni post-test. D, MCF7 cells transiently co-transfected with ERRE-luciferase and AIB1, ERRβsf, ERRβ2, or a 1:1 ratio of AIB1:ERRβsf or AIB1:ERRβ2 for ~24 h. N = 3 for a representative assay shown in triplicate, two-way ANOVA with Bonferroni post-test.

The estrogen-related receptor (ERR) family has direct, DNA binding-associated transcriptional activity at a number of promoter elements, including the estrogen-related response element (ERRE) half site, classical estrogen response elements (EREs), and a hybrid ERE/ERRE element [29–32]. These receptors have also been implicated in indirect transcriptional control through tethering to AP1 [33] and SP1 [34] transcription factors. We recently published that ERRβsf has constitutive and ligand-modulated activity on the p21 promoter in cellular models of glioblastoma (GBM, [27]) but that ERRβ2 cannot activate the p21 promoter-reporter. Here, we measured the activity of these exogenous splice variants on ERE- and ERRE-luciferase heterologous promoter-reporter constructs in breast cancer cells. In estrogen receptor alpha-negative (ER-) MDA-MB-231 cells, exogenous ERRβsf has robust basal transcriptional activity that is equally enhanced by exposure to two ERRβ/γ synthetic agonists: DY131 (DY) or GSK4716 (GSK, Figure 1A). ERRγ is also active under basal and ligand-stimulated conditions, but ERRβ2 has no effect on ERE-luciferase expression. In estrogen receptor alpha-positive (ER+) MCF7 cells, a similar trend is observed for ERRE-luciferase; ERRβsf and ERRγ both show basal and ligand-induced transcriptional activity in response to DY131, while ERRβ2 does not (Figure 1B). We showed previously that ERRβ2 is a dose-dependent dominant-negative inhibitor of ERRβsf on the p21 promoter in GBM cell lines [27]. Here, ERRβ2 behaves similarly on the ERRE-luciferase construct in MCF7 breast cancer cells (Figure 1C), where its exogenous expression also significantly represses background ERRE activity.

Several molecular mechanisms could explain ERRβ2's dominant-inhibitory activity. Ligand-regulated and orphan nuclear receptors both rely heavily on partner proteins – coactivators and corepressors – to confer specificity upon their transcriptional activity [35]. While most studies of ERR coactivators have focused on PGC1α and β (e.g. [17]), ERRβsf requires nuclear receptor coactivator 3 (NCOA3 or AIB1) to perform transcription-dependent functions in mouse embryonic stem cell self-renewal [21], and cooperates with AIB1 to modulate G1/S checkpoint integrity in this context [23, 24]. Here, we show that exogenous expression of AIB1 [36] alone can induce ERRE-luciferase activity in MCF7 cells, and enhance ERRβsf-mediated activation of the ERRE-luciferase reporter in the absence of ligand (Figure 1D). By contrast, ERRβ2 significantly inhibits AIB1-mediated induction of ERRE-luciferase activity. These data show that the ERRβ2 splice variant has no transcription factor activity and serves as a dominant-negative inhibitor of ERRβsf-dependent transcription in breast cancer. They further suggest that this is due, at least in part, to competition for coactivators.

### ERRβ/γ agonist DY131 is growth-inhibitory in breast cancer cells

The role of ERRβ and its splice variants in breast cancer is not fully defined. High levels of total ESRRB mRNA in primary breast tumors are associated with a reduction in cells in S phase and increased expression of estrogen receptor beta (ERβ) [37]. In exogenous expression studies, ERRβsf interacts with ER, alters the intranuclear localization of ER, and suppresses ER-mediated gene transcription in MCF7 cells [38]. Exogenous expression of ERRβ “long form” (a synonym for ERRβ2 used by some) in MCF7 cells leads to apoptosis and also shows an interaction with ER that is attenuated upon exposure to estradiol [39]. ERRγ's role in breast cancer is complex and context-dependent; while several studies show an association between this receptor and indicators of good prognosis [37] or directly demonstrate a growth-suppressive role [40, 41], others report that it can promote estrogen-independent and Tamoxifen-resistant growth [42–46], and metabolically reprogram non-transformed mammary epithelial cells to successfully adapt to anoikis [47].

**Figure 2:**
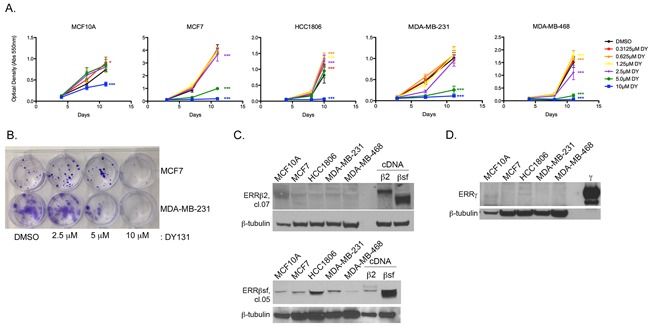
ERRβ/γ agonist DY131 is growth-inhibitory in breast cancer cells **A.** Crystal violet staining of breast cancer and non-transformed breast epithelial cell lines in the presence or absence of the indicated concentrations of DY131 or DMSO control over time. N = 6 for a representative assay performed in sextuplicate, two-way ANOVA with Bonferroni post-tests in each cell line vs. DMSO control at Day 10/11. **B.** Clonogenic survival assay for MCF7 and MDA-MB-231 cells seeded at low density and cultured for 13 d after a single, 24 h exposure to the indicated concentrations of DY131. **C-D.** Representative Western blot analysis of basal ERRβsf, ERRβ2, and ERRγ expression in non-transformed mammary epithelial and breast cancer cells. β2 and βsf positive controls are from MDA-MB-231 cells transiently transfected with the indicated cDNA. γ positive control is purified protein.

We therefore tested the capacity of the ERRβ/γ agonist DY131 to modulate breast cancer and non-transformed mammary epithelial cell growth, as measured by crystal violet staining (Figure 2A, Supplementary Figure S1A). In addition to ER+ MCF7 cells and ER- MDA-MB-231 cells representative of the mesenchymal stem-like subtype of triple negative breast cancer (TNBC) [48], we used two additional TNBC cell lines – HCC1806 (basal-like 2) and MDA-MB-468 (basal-like 1) – in addition to the MCF10A non-transformed mammary epithelial cell line. All cancer cell lines are completely growth-inhibited by the highest concentration (10 μM) of DY131, while MCF7, MDA-MB-231, and MDA-MB-468 are also significantly inhibited by 5 μM, and MCF7 and MDA-MB-468 cells by 2.5 μM. MCF10A cells are only modestly growth-inhibited by 10 μM DY131, and responsiveness is not fully explained by differences in the basal (untreated) growth rate of the cell lines (Supplementary Figure S1B), suggesting that DY131 preferentially inhibits the growth of cancer cells. In clonogenic survival assays, MDA-MB-231 show a dose-dependent reduction in colony formation after a single, 24 h exposure to DY131 (Figure 2B), while MCF7 cells show reduced colony formation at 10 μM. We then measured expression of two endogenous ERRβ splice variants – ERRβsf and ERRβ2 – in these cell lines (Figure 2C) using a pair of monoclonal antibodies that preferentially detect each endogenous splice variant [27]. Both antibodies detect exogenous/overexpressed receptor, as demonstrated by the positive controls (cells transfected with the indicated cDNA). ERRβ2 and ERRβsf are expressed in all cell lines. We previously reported [42] that MCF7 cells express very low to undetectable levels of ERRγ. We confirm this finding here, and show that this is also true for MDA-MB-231 and MDA-MB468 cells, while MCF10A and HCC1806 cells express some ERRγ (Figure 2D). In summary, multiple breast cancer cell lines are preferentially growth-inhibited by the ERRβ/γ agonist DY131 as compared to a non-transformed breast epithelial cell line, and all express detectable levels of ERRβsf and ERRβ2 protein, whereas ERRγ protein expression is not consistently observed.

### DY131 induces apoptotic cell death

**Figure 3:**
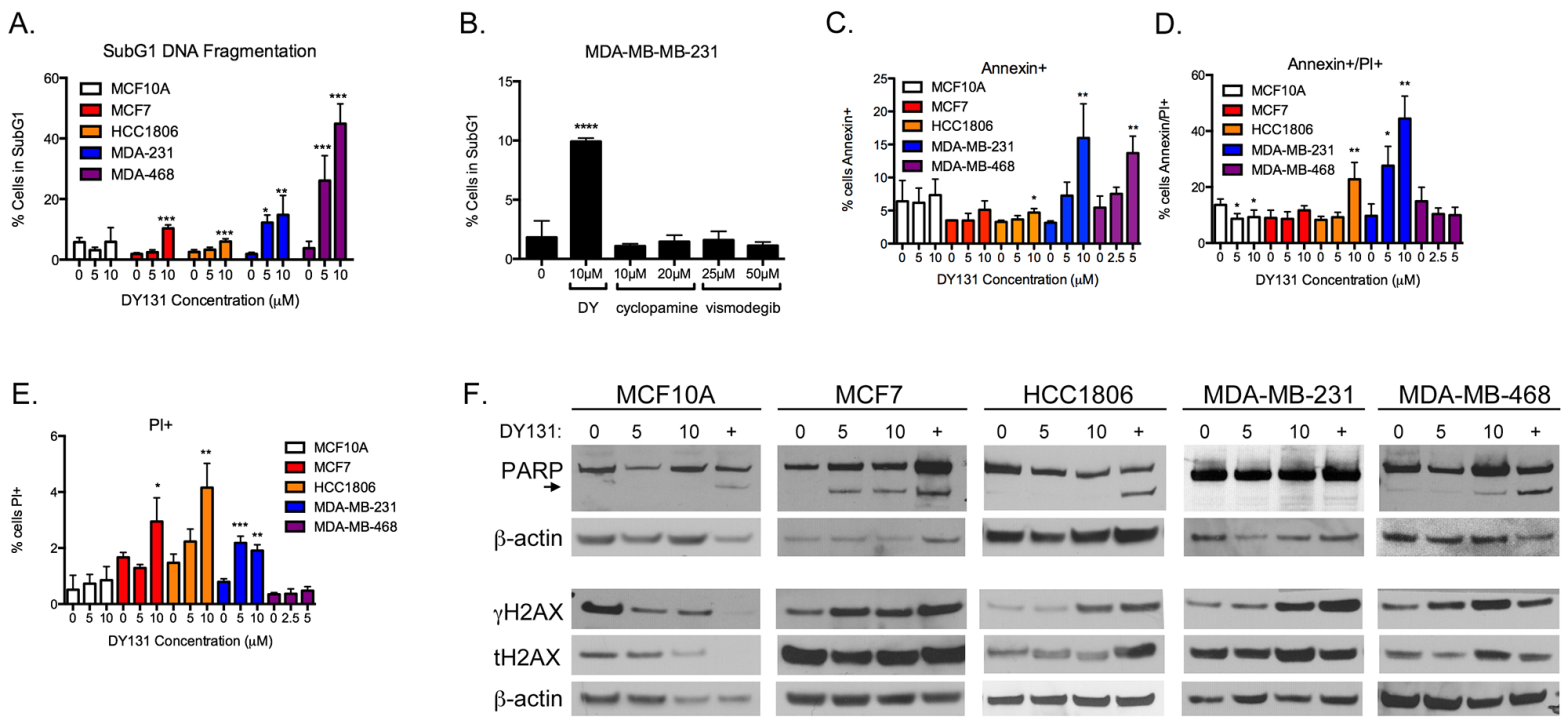
DY131 induces apoptotic cell death **A.** Percent of cells exhibiting fragmented DNA (subG1 DNA content as measured by propidium iodide staining of fixed cells) after exposure to DY131 for 24 h as determined by flow cytometry. N = 3 – 5 independent assays, one-way ANOVA with Tukey's post-test. **B.** Effect of Smoothened inhibitors cyclopamine and vismodegib on subG1 DNA content in MDA-MB-231 cells after 24 h exposure. N = 3 independent assays, one-way ANOVA with Tukey's post-test. **C-E.** Percent of cells staining positive for cell-surface Annexin V and/or propidium iodide uptake by live cells after exposure to DY131 for 12-24 h as determined by flow cytometry. N = 3 – 5 independent assays, one-way ANOVA with Tukey's post-test. **F.** Representative Western blot analysis of PARP, γH2AX, and total H2AX in DY131-treated cells (24 h). + denotes doxorubicin positive control (24 h). Arrowhead indicates PARP cleavage product.

To determine whether the growth-inhibitory activity of DY131 can be attributed to cytotoxic (cell killing) activity, all 5 cell lines were treated with increasing concentrations of DY131 and analyzed for fragmented (subG1) DNA content as a measure of cell death (Figure 3A). All breast cancer cell lines show a significant, dose-dependent increase in the subG1 fraction, with MDA-MB-231 and MDA-MB-468 cells being the most sensitive, whereas non-transformed MCF10A cells are unaffected by DY131 treatment. DY131 has been reported to have off-target activity through direct inhibition of Hedgehog signaling by binding to Smoothened via the same mechanism as more conventional inhibitors (cyclopamine and vismodegib, [49]), and in prostate cancer cells exogenous ERRβsf can regulate Hedgehog target genes [50]. However, it is well-established that non-transformed MCF10A, MCF7, and MDA-MB-231 cells lack expression of Smoothened and are relatively resistant to cell killing by Smoothened inhibitors [51–53]. Consistent with this, neither cyclopamine nor vismodegib phenocopies DY131-induced cell death in MDA-MB-231 cells (Figure 3B). Smoothened-independent mechanisms of Hedgehog pathway signaling can include activation of the transcription factor GLI Family Zinc Finger 1 (GLI1) [54], so we tested whether DY131 could inhibit GLI1 transcription factor activity in MDA-MB-231 cells (Supplementary Figure S2). Exogenous GLI1 activity is modestly inhibited by DY131, though not to the same extent as by the direct GLI inhibitor arsenic trioxide (ATO [54]). Therefore, DY131 may have Hedgehog pathway-inhibitory activities in some cellular contexts, but our data suggest that this mechanism of action is not a key contributor to DY131-induced cell death in breast cancer cell lines.

To better define the cellular mechanism of death induced by DY131, we stained live cells for Annexin V surface expression and propidium iodide uptake (Figure 3C–3E). There is a significant, three to four-fold increase in Annexin V single-positive cells upon DY131 treatment in MDA-MB-231 and MDA-MB-468 cells, indicative of early-stage apoptosis. MCF7 cells show no early apoptosis, while HCC1806 cells show a small, statistically significant increase in early apoptosis. HCC1806 cells exhibit significantly increased double-positive and propidium iodide single-positive staining associated with late-stage apoptosis and necrosis, respectively. MCF7 cells show a modest (though statistically significant) increase in propidium iodide single-positive cells in the presence of 10 μM DY131. By contrast, DY131 does not induce any stage of apoptosis in MCF10A cells.

In our prior study of cellular models of GBM, we showed that DY131-induced apoptosis is accompanied by cleavage of poly ADP-ribose polymerase (PARP) [27], a well-characterized substrate of executioner caspases [5]. MCF7, MDA-MB-231, and MDA-MB-468 cells exhibit PARP cleavage in response to DY131 (Figure 3F), while HCC1806 and MCF10A cells do not. All cells display PARP cleavage upon treatment with the doxorubicin positive control. We also observe an increase in Ser139 phosphorylation of histone H2AX (γH2AX), a histone modification that can accompany DNA damage, apoptosis, X chromosome inactivation, and/or mitosis (reviewed in [55, 56]) in the four breast cancer cell lines, but not MCF10A cells.

### DY131 does not induce a conventional DNA damage response or bind DNA directly

**Figure 4:**
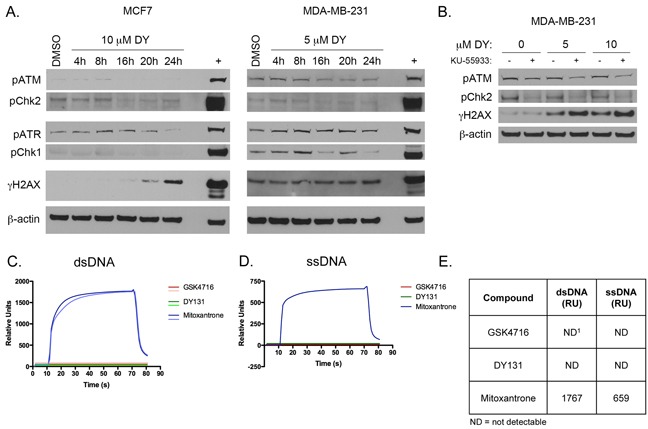
DY131 does not induce a conventional DNA damage response or bind DNA directly **A.** Representative time course Western blot analysis of DNA damage response kinases and γH2AX in response to DY131 in MCF7 and MDA-MB-231 cells. + denotes doxorubicin positive control (24 h). **B.** Representative Western blot analysis of ATM signaling pathway activation and γH2AX in MDA-MB-231 cells in response to DY131 following a 1 h pre-treatment with ATM inhibitor KU-55933. **C-D.** Surface plasmon resonance (BIAcore) sensogram of DY131, GSK4716, or mitoxantrone positive control binding to dsDNA or ssDNA. **E.** Table summarizing results of BIAcore binding studies. Values shown are the peak Relative Unit (RU) values after 60 s injection of compound. ND^1^ = not detectable. Data shown are from a representative experiment, performed twice.

The induction of γH2AX by DY131 raises the possibility that this compound can elicit a DNA damage response (DDR). In the context of the DDR, rapid H2AX phosphorylation is catalyzed by ataxia telangiectasia mutated (ATM) or ataxia telangiectasia and Rad3-related (ATR) at double-strand breaks (DSBs). However, a DY131 time course in MCF7 and MDA-MB-231 cells shows no activation of this signaling cascade in contrast to the doxorubicin positive control, and the induction of γH2AX is not detected until later time points (≥20 h, Figure 4A). Furthermore, pre-treatment with the ATM inhibitor KU-55933 does not prevent DY131-induced γH2AX (Figure 4B). We subsequently performed a surface plasmon resonance (BIAcore) experiment with DY131 and the related ERR agonist GSK4716 to determine whether these compounds could bind double strand (ds) or single strand (ss) DNA directly (Figure 4C–4E). While the mitoxantrone positive control readily binds dsDNA and ssDNA, neither ERR agonist is competent to do so. Altogether, these data support the conclusion that DY131-induced γH2AX is not associated with direct DNA damage or nucleic acid binding.

### DY131 induces G1 and G2/M cell cycle arrest

**Figure 5:**
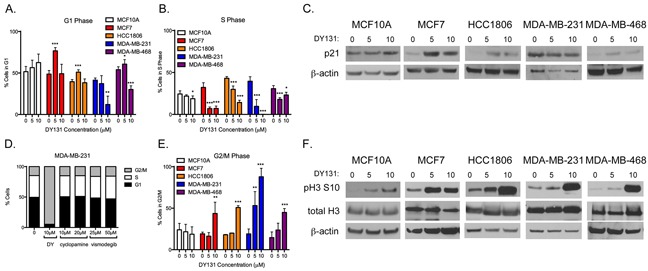
DY131 induces G1 and G2/M cell cycle arrest **A.** Percent of cells in the G1 phase of the cell cycle after exposure to DY131 for 24 h as determined by flow cytometry. N = 3 – 5 independent assays, one-way ANOVA with Tukey's post-test. **B.** Percent of cells in S phase of cell cycle after exposure to DY131 for 24 h as determined by flow cytometry. N = 3 – 5 independent assays, one-way ANOVA with Tukey's post-test., **C.** Representative Western blot analysis of p21 in DY131-treated cells (24 h). **D.** Effect of Smoothened inhibitors cyclopamine and vismodegib on the cell cycle profile of MDA-MB-231 cells after 24 h exposure. Data shown are from a representative experiment, performed three times. **E.** Percent of cells in the G2/M phase of the cell cycle after exposure to DY131 for 24 h as determined by flow cytometry. N = 3 – 5 independent assays, one-way ANOVA with Tukey's post-test. **F.** Representative Western blot analysis of phosphorylated Serine 10 and total Histone H3 in DY131-treated cells (24 h).

Prior studies by our group [27] and others [25, 57] have shown that ERRβsf or ERRγ can induce G1 cell cycle arrest, while ERRβ2 mediates a G2/M arrest in response to DY131 [27]. Here, we show that MCF7, HCC1806, and MDA-MB-468 undergo a significant G1 arrest at 5 μM (Figure 5A), while MDA-MB-468 cells show minimal arrest and MDA-MB-231 cells show none. There is a dose-dependent trend towards G1 arrest in MCF10A cells, but this is not statistically significant. All cell lines show a significant reduction in S phase fraction (Figure 5B), with MCF10A cells again the least affected. The magnitude of G1 arrest is reflected in a corresponding increase in p21 expression (Figure 5C), which is not p53-dependent since MDA-MB-468 cells have mutated p53 and HCC1806 cells are p53 null [58]. Neither Smoothened inhibitor causes G1 arrest or a reduction in S phase in MDA-MB-231 cells (Figure 5D).

The four breast cancer cell lines all exhibit significant G2/M arrest in the presence of 10 μM DY131, with MDA-MB-231 cells also showing a significant, dose-dependent G2/M arrest at 5 μM (Figure 5E). Consistent with this, in all breast cancer cell lines we observe a strong increase in Ser10 phosphorylation of histone H3 (Figure 5F), a histone modification associated with chromatin condensation in prophase [59–61], as well as immediate early gene transcription [62]. Neither a G2/M arrest nor a robust increase in Ser10 phosphorylation of histone H3 occurs in non-transformed MCF10A cells treat at the same concentrations of DY131. In summary, DY131 induces a bimodal cell cycle arrest in MCF7, HCC1806, and (to some extent) MDA-MB-468 cells, but only G2/M arrest in MDA-MB-231 cells.

### DY131-induced p38 MAPK activity is required for cell death, but not cell cycle arrest

One potential explanation for DY131-induced changes in H3 Ser10 phosphorylation and/or γH2AX in the absence of a conventional DDR is activation of the p38 mitogen-activated protein kinase (MAPK) cascade. p38 can directly phosphorylate H2AX *in vitro* [63] and is responsible for apoptosis-associated *in vivo* γH2AX induction either directly or through activation of downstream kinases such as mitogen-activated protein kinase activated kinase 2 (MAPKAPK2) [64, 65]. Similarly, p38 can phosphorylate H3 Ser10 directly *in vitro* [66], as can the p38 substrate mitogen- and stress-activated protein kinase 1 (MSK1) [62].

**Figure 6:**
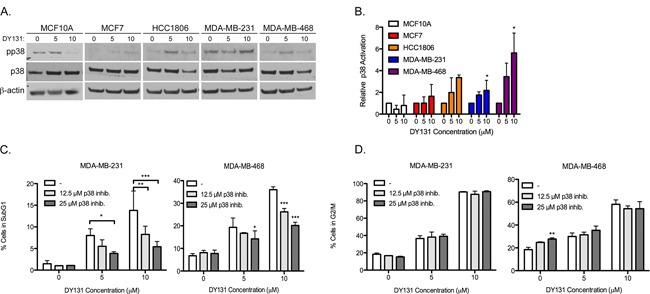
DY131-induced p38 MAPK activity is required for cell death, but not cell cycle arrest **A.** Representative Western blots for activating phosphorylation of p38 in DY131-treated cells. **B.** Densitometry analysis of the ratio of phosphorylated to total p38 relative to β-actin are normalized to the level of the DMSO control for each cell line. N = 3 independent assays, one-way ANOVA with Tukey's post-test. **C.** Percent of cells exhibiting fragmented DNA (subG1 DNA content as measured by propidium iodide staining of fixed cells) after a 1 h pre-treatment with p38 inhibitor SB203580 before exposure to DY131 for an additional 24 h as determined by flow cytometry. N = 3 independent assays, two-way ANOVA with Bonferroni post-test. **D.**, Percent of cells in the G2/M phase of the cell cycle after a 1 h pre-treatment with p38 inhibitor SB203580 before exposure to DY131 for an additional 24 h as determined by flow cytometry. N = 3 independent assays, two-way ANOVA with Bonferroni post-test.

Activating phosphorylation of p38 is weak or absent in MCF10A and MCF7 cells treated with DY131 (Figure 6A (Western blot) and 6B (densitometry)). By contrast, HCC1806 show a trend towards p38 phosphorylation, while MDA-MB-231 and MDA-MB-468 cells show a significant, two to six-fold induction in p38 phosphorylation at 10 μM. Because the latter two cell lines are also the most responsive to DY131-induced G2/M arrest and cell death, we pretreated them with the inhibitor SB203580 to test p38's contribution to these phenotypes. Pharmacological p38 inhibition significantly and dose dependently reduces DY131-induced subG1 (cell death) in both cell lines (Figure 6C), but does not inhibit DY131-mediated G2/M arrest (Figure 6D). Altogether, these data show that DY131 activates p38 in breast cancer cells, and while this plays a key role in drug-induced cell death, it is not required for G2/M arrest.

### ERRβ2 promotes DY131-induced histone H3 phosphorylation

**Figure 7:**
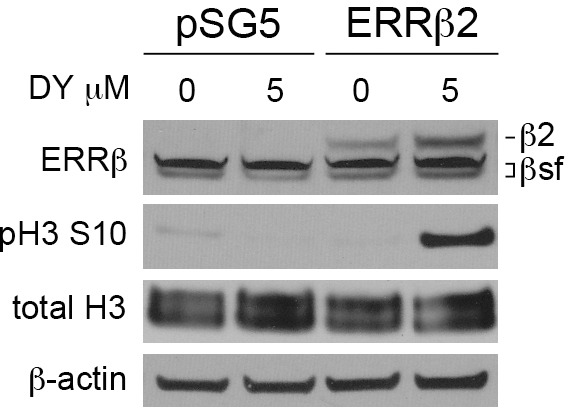
ERRβ2 promotes DY131-induced histone H3 phosphorylation Representative Western blot analysis of ERRβ2, phosphorylated Serine 10 and total Histone H3 in MCF7 cells transiently transfected with either ERRβ2 or pSG5 empty vector, then treated with DY131 or DMSO control for 18-20 h. Exogenous ERRβ2 expression was detecting using H6705 (cl.05) in order to also visualize endogenous ERRβsf.

Because our prior studies in GBM have shown that exogenous ERRβ2 promotes DY131-mediated G2/M arrest [27], we tested whether this is also true in breast cancer. We selected the cell line with the strongest DY131-induced G1 arrest at 5 μM (MCF7, see Figure 5A) in which to test whether exogenous ERRβ2 can induce markers of G2/M arrest. MCF7 cells transiently transfected with exogenous ERRβ2 (visualized using the cl.05 antibody so as to also show endogenous ERRβsf) show a strong increase in Ser10 phosphorylation of histone H3 (Figure 7). We could not determine whether exogenous ERRβ2 suppresses DY131-mediated G1 arrest as measured by a reduction in p21, because in these cells transient transfection, even with the empty vector, artificially increases basal p21 levels such that DY131-mediated induction is no longer observable (not shown).

### DY131 delays chromosome segregation in mitosis

**Figure 8:**
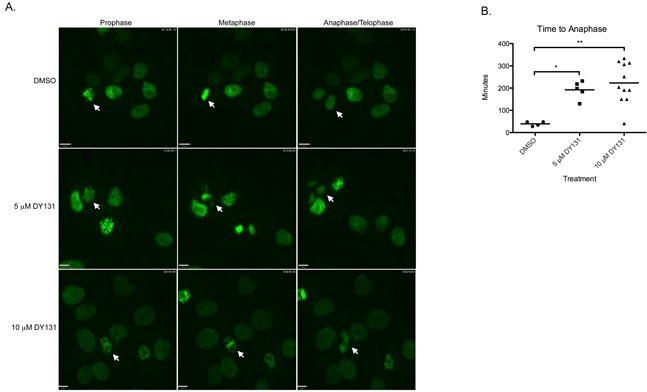
DY131 delays chromosome segregation in mitosis **A.** Individual frames representative of prophase, metaphase, and anaphase/telophase from live-cell confocal microscopy of MCF7 cells stably expressing GFP-H2B. Cells were accumulated in G2 by exposure to nocodazole, then released into DY131 or DMSO control. Arrows denote cells of interest. **B.** Quantitation of time elapsed from chromatin condensation (prophase) to anaphase in MCF7 cells stably expressing GFP-H2B after release from nocodazole block into DY131 or DMSO control. N = 4 – 11 cells, one-way ANOVA with Tukey's post-test.

Our data demonstrating DY131-induced G2/M cell cycle arrest, coupled with DY131-mediated induction of histone H3 Ser10 phosphorylation that is potentiated by exogenous ERRβ2, are indicative of an early (pre-anaphase) mitotic defect, but a more precise definition of where DY131 can perturb mitosis is required. We therefore performed live-cell confocal video microscopy of MCF7 cells stably transfected with H2B-GFP [67]; these cells were used for this experiment because although they are aneuploid, most contain a single nucleus, which enables semi-automated tracking of mitotic progression [68]. Cultures were enriched for cells with G2 DNA content by exposure to nocodazole, and then released into media containing DMSO control or DY131 (Figure 8A). As a control, we also tested two different compounds with known effects on mitotic progression. The cyclin-dependent kinase inhibitor flavopiridol accelerates mitotic exit, leading to a pseudo G1-like state with >4n DNA content [69], while the microtubule stabilizer paclitaxel halts cells in prophase [70]. We observe both of these phenotypes in MCF7 cells (Supplementary Movies 1 and 2). Nocodazole-treated cells released into DMSO show typical mitotic transit, with a mean time of progression from prophase to anaphase of ~39 minutes (Figure 8B, Supplementary Movie S3). By contrast, release into DY131 causes a significant, dose-dependent delay in mitotic progression in those cells that do divide (Supplementary Movies S4 and S5). Thirty-three percent (33%) of cells in 5 μM remain pre-anaphase, while 66% eventually divide to enter the next G1 phase. Of cells in 10 μM DY131, 35% do not progress to anaphase, and 65% eventually divide. DY131-exposed cells also exhibit a more disorganized metaphase plate with evidence of lagging chromosomes (Figure 8A, white arrowheads in metaphase panels from DY131-treated cells). These data suggest that DY131 delays mitotic progression from prophase to anaphase by causing errors in chromosome segregation.

### DY131 causes monopolar and multipolar spindles

**Figure 9:**
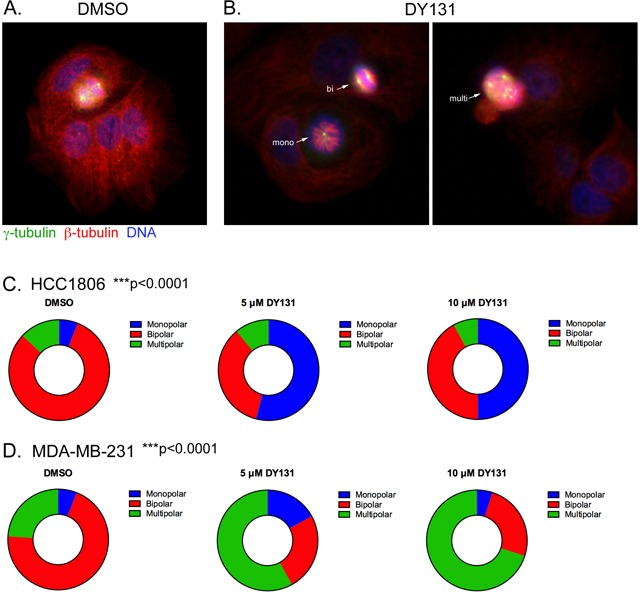
DY131 causes monopolar and multipolar spindles **A.** and **B.** HCC1806 were treated with DMSO and DY131 (5 uM) for 24 h. The cells were fixed and stained for the centrosomal marker γ-tubulin, β-tubulin and DAPI. **C.** and **D.** Graphical representation of the fraction of mono-, bi- and multipolar spindles for HCC1806 and MDA-MB-231 cells treated with DY131 or DMSO control for 24 h. N = 3 independent assays, chi squared test.

Delays in mitotic progression and lagging chromosomes caused by acute exposure to DY131 prompted us to test whether longer treatment causes mitotic catastrophe by disrupting centrosome number and/or spindle polarity. HCC1806 cells were treated with DY131 or DMSO for 24 h, fixed, and stained for the centrosomal marker γ-tubulin, β-tubulin and DNA (Figure 9A–9C). While DMSO-treated mitotic cells contain two clearly separated γ-tubulin puncta indicative of properly oriented centrosomes flanking metaphase chromosomes, most DY131-treated HCC1806 cells have monopolar spindles indicative of 1 centrosome per cell and more disorganized metaphase plates or none at all. By contrast, MDA-MB-231 cells have significantly more multipolar spindles, with ≥3 centrosomes per cell following DY131 treatment (Figure 9D). Altogether, these data suggest that DY131 treatment leads to catastrophic mitotic spindle defects that are likely responsible for the observed DiM.

### Endogenous ERRβ2 localizes to the cytosol and centrosomes

**Figure 10:**
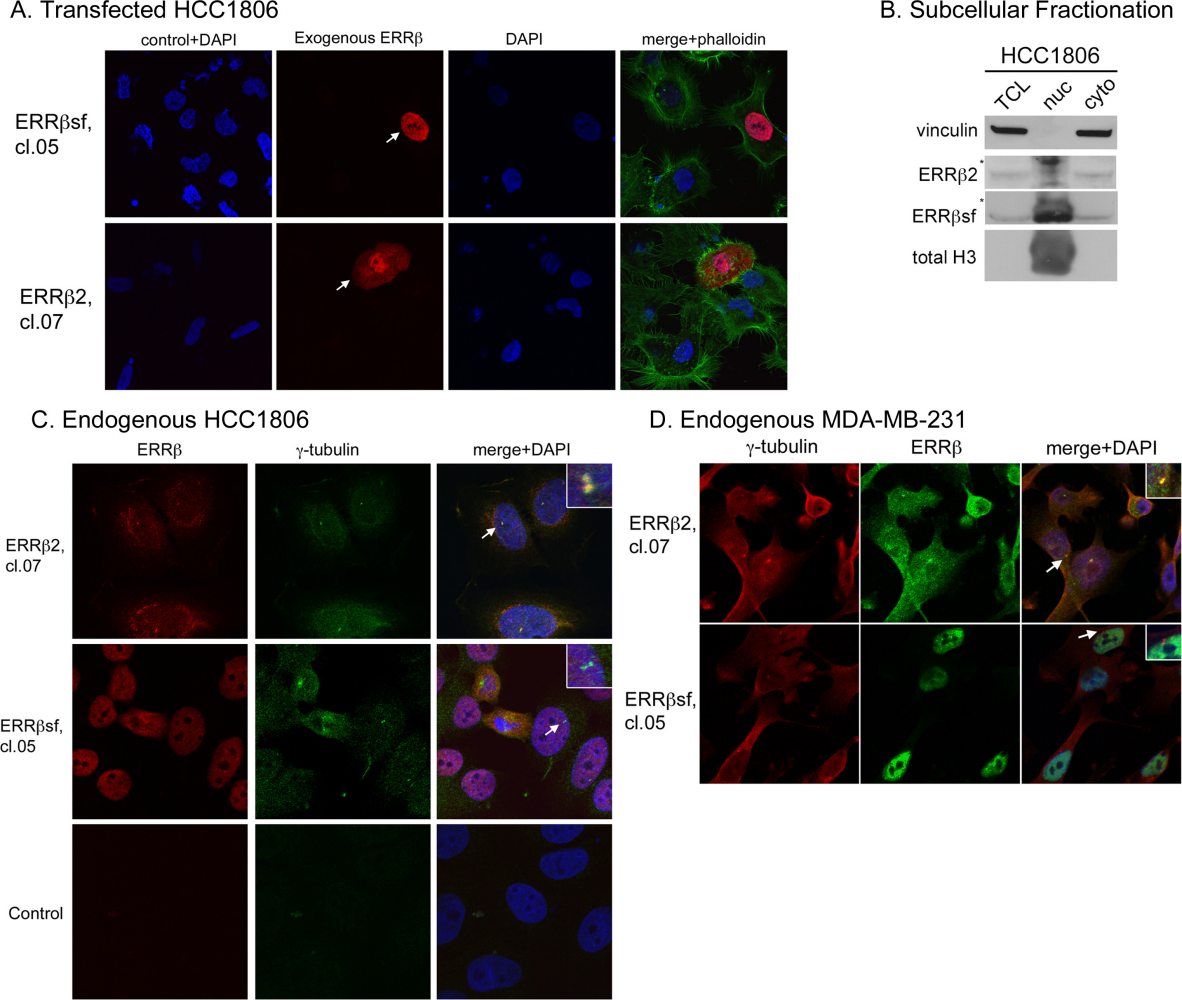
Endogenous ERRβ2 localizes to the cytosol and centrosomes **A.** Subcellular localization of exogenous ERRβsf (cl.05, top) and ERRβ2 (cl.07, bottom) together with DNA (DAPI) in HCC1806 cells transfected with the appropriate cDNA. Arrowheads denote transfected cells. Control + DAPI indicates cells stained only with secondary antibodies plus DAPI. Phall = phalloidin. **B.** REAP fractionation of HCC1806 cells followed by Western blot analysis of vinculin, ERRβ2, ERRβsf, and total Histone H3. * denotes nonspecific band in nuclear extracts. Lanes were loaded as follows: TCL, 40 μl; nuc, 20 μl; cyto, 40 μl. **C.** Subcellular localization of endogenous ERRβ2 (top panels) or ERRβsf (bottom panels) together with the centrosome marker γ-tubulin and DNA (DAPI) in HCC1806 cells. Insets show an expanded view of centrosomes identified by arrows. Control indicates cells stained only with secondary antibodies plus DAPI. **D.** MDA-MB-231 cells, same as in C.

Zhou et al. showed in COS-1 cells that exogenous ERRβsf is a nuclear protein, while exogenous ERRβ2 is primarily found in the cytosol [26]. In HCC1806 cells transfected with these cDNAs and stained using splice variant-preferential monoclonal antibodies (cl.05 for ERRβsf and cl.07 for ERRβ2), ERRβsf is localized to the nucleus, while ERRβ2 is found in both the nucleus and cytosol (Figure 10A). We then used biochemical and imaging approaches to determine whether endogenous ERRβsf and ERRβ2 are also localized to discrete cellular compartments. Subcellular fractionation shows that ERRβ2 is abundant in the cytosolic fraction, while ERRβsf is enriched in the nuclear pellet (Figure 10B). Confocal immunofluorescence microscopy for endogenous ERRβ expression in HCC1806 (Figure 10C) and MDA-MB-231 breast cancer cells (Figure 10D) confirms that ERRβsf is found in the nucleus of both cell lines. However, endogenous ERRβ2 exhibits diffuse staining throughout the cell with statistically significant enrichment in bright puncta that colocalize with γ-tubulin (upper right portion of each panel, arrows and inset of 10C and 10D, and Supplementary Figure S3). Centrosomal localization of endogenous ERRβ2 is consistent with delayed mitotic progression and the spindle defects observed upon DY131 treatment.

## DISCUSSION

Liganded receptors have long been successful drug development targets, with selective estrogen and androgen receptor modulators key to the clinical management of hormone receptor-positive breast and prostate cancer for decades. The recent discovery that liver-X nuclear hormone receptor agonists are highly effective against metastatic melanoma [71] underscores the potential clinical utility of small molecules that target this and other [72] orphan nuclear receptors. Here we present evidence that i. a synthetic ligand for estrogen-related receptors β and γ (DY131) is growth-inhibitory towards breast cancer cells, ii. the ERRβ2 splice variant is a cytosolic and centrosomal protein, and iii. DY131-mediated anti-mitotic activity is characterized by spindle polarity defects.

DY131 induces cell death in hormone receptor-positive and –negative breast cancer cell lines, while it has no significant cytotoxic effect on MCF10A non-transformed mammary epithelial cells (Figure 3). The cell death phenotype is characterized by features of apoptosis (Annexin V membrane staining and PARP cleavage). It is also accompanied by the induction of γH2AX, historically an indicator of DNA double-strand breaks (DSBs) that has more recently emerged as an alternate measure of apoptosis [55, 56, 73]. γH2AX as a marker for DY131-mediated apoptotic cell death rather than DNA damage is supported by two specific pieces of evidence: the lack of involvement of DNA damage-responsive kinases (ATM, ATR; (Figure 4)); and the requirement for an active p38 MAPK pathway (Figure 6), of which γH2AX is a known target [63–65].

In the present study, three of four breast cancer cell lines show a bimodal cell cycle arrest in response to DY131 (G1 at a lower concentration, G2/M at a higher concentration), with MDA-MB-231 cells exclusively undergoing G2/M arrest (Figure 5). Our studies in GBM [27] were the first to establish that the ERRβsf and ERRβ2 splice variants have distinct cell cycle regulatory roles, with ERRβsf mediating G1 arrest and the ERRβ2 isoform responsible for DY131-induced G2/M arrest. Here, we are able to shift the cell cycle arrest phenotype in MCF7 cells from G1 to G2/M at a lower concentration of DY131 through exogenous expression of ERRβ2, as measured by an increase in Ser10 phosphorylation of histone H3 (Figure 7). These data further support the requirement for ERRβ2 in drug-induced G2/M arrest. In GBM, we showed that ERRβsf is a transcriptionally active receptor that drives expression of p21, while ERRβ2 has no transcription factor activity and acts in a dominant-inhibitory fashion on the p21 promoter [27]. Here, we show that in breast cancer cells, ERRβ2 similarly cannot drive promoter-reporter luciferase activity and suppresses ERRβsf-driven transcription of ERRE-enhanced promoter-reporter constructs, in part through competition for coactivators like AIB1 (Figure 1). However, the mechanism(s) underlying the bimodal arrest profile in some breast cancer cells, which we did not observe in GBM, is/are currently unknown. One hypothesis is that in breast cancer, there is a dose-dependent switch in splice variant dominance from ERRβsf to ERRβ2 owing to changes in the abundance of receptors and/or coregulatory proteins. A more likely alternative is that ERRβ2 is the dominant splice variant at all times and the G1 arrest observed at lower concentrations of DY131 is due to mitotic slippage, while at higher concentrations the drug-induced block of mitotic transit is more robust, resulting in G2/M arrest and DiM. Mitotic slippage can be attributed to a failure of the spindle assembly checkpoint (SAC) and/or the anaphase promoting complex/cyclosome (APC/C-Cdh1, e.g. [74]). Our observation that treatment of nocodazole-released MCF7 cells with 5 μM and 10 μM DY131 causes the appearance of lagging chromosomes in metaphase (Figure 8) is suggestive of a drug-induced SAC and/or APC/C-Cdh1 defect.

Two novel findings of the present work are that endogenous ERRβ2 is a cytosolic protein that can localize to centrosomes, and treatment of breast cancer cells with DY131 leads to spindle polarity defects. Prior studies have evaluated the subcellular localization of untagged and epitope-tagged ERRβ splice variants in breast cancer and other cell lines, and have consistently shown that exogenous ERRβsf is a nuclear protein [26, 38], which we also observe for the endogenous splice variant in MDA-MB-231 and HCC1806 cells. However, there are discrepancies in the reported subcellular localization of exogenous ERRβ2. Zhou et al. show broad nuclear and cytoplasmic localization of untagged ERRβ2 in most COS-1 cells, with only ~30% cells showing predominantly nuclear staining [26]; this is similar to what we observe in HCC1806 cells transfected with exogenous ERRβ2, as well as endogenous ERRβ2 in HCC1806 and MDA-MB-231 cells (Figure 10). By contrast, Bombail et al. [75] and Sengupta et al. [39] report exclusive nuclear localization of yellow fluorescent protein (YFP)-tagged “long form” receptor (ERRβ2) in ovarian and breast cancer cells, respectively. We propose that these discordant results are due to altered localization of epitope tagged receptor, since we also observe that a SNAP-ERRβ2 fusion protein is only found in the nucleus (not shown). In MDA-MB-231 and HCC1806 breast cancer cells we observe endogenous ERRβ2 throughout the cytosol and in centrosomes, as shown by colocalization with γ-tubulin puncta adjacent to the nucleus. In addition, centrosome visualization (Figure 10C and 10D, Supplementary Figure S3) requires harsher post-permeabilization strategies like methanol [76], which may explain why ERRβ2 expression in this compartment has not been previously reported and why it is not evident in Figure 10A. To our knowledge, the only other orphan nuclear receptor that localizes to centrosomes is NR5A1 (steroidogenic factor-1, (SF1)), where its silencing causes centrosome duplication and chromosomal instability through a transcription-independent mechanism involving DNA-PK [77–79]. By contrast, exposure of MDA-MB-231 and HCC1806 cells to DY131 leads to the appearance of multipolar and monopolar spindles, respectively.

The ability of DY131 to induce spindle defects (Figure 9) and DiM positions ERRβ, particularly the ERRβ2 splice variant, as a novel therapeutic target. Many aneuploid cells have more than two centrosomes, referred to as amplified or supernumerary centrosomes, and it is estimated that ~80% of invasive breast tumors display this phenotype [80]. Centrosome amplification is common in cellular models of HER2-enriched breast cancer, and in this setting can also drive cancer cell invasion and migration [81, 82]. However, multipolar spindles do not support proper bipolar division, and their presence can cause chromosome missegregation and cell death during or immediately following mitosis [83, 84]. This has recently been shown to be the primary *in vivo* mechanism for paclitaxel-induced breast cancer cell death [85]. To avoid apoptosis triggered by centrosome amplification, cancer cells have evolved ways to cluster or group these extra centrosomes together during cell division [86]. These clustering mechanisms also present a therapeutic opportunity, and centrosome declustering drugs have been proposed as alternatives to conventional antimitotic therapies in breast and other cancers [8–11]. Whether DY131 is a centrosome declustering agent or leads to the appearance of multipolar spindles by other means (or both) remains to be determined. MDA-MB-231 cells have supernumerary centrosomes and are sensitive to known centrosome declustering agents (e.g. [10, 11]), but only ~35% of a given population of these cells have clustered centrosomes [86]. It should also be noted that many centrosome declustering drugs can also cause centrosome amplification [10]. Further studies will be required to establish precisely how DY131 causes spindle defects, and why this can manifest as either multipolar or monopolar spindles.

Centrosome amplification is known to associate with poor outcome in TNBC [87], and we postulate that if ligand-mediated activation of ERRβ2 leads to mitotic arrest, spindle polarity defects, and DiM in cell line models of TNBC, expression of this splice variant should correlate with improved outcome in TNBC clinical specimens. We performed meta-analysis [88] of publicly available gene expression data from ER-/HER2- clinical specimens classified as basal [89] arrayed on the Affymetrix U133 Plus 2.0 platform, which has two ESRRB probesets that detect different combinations of transcript variants (Supplementary Figure S4). The probeset corresponding to a third splice variant not specifically studied here (ERRβ-Δ10) is not significantly associated with improved recurrence free survival (RFS) and distant metastasis free-survival (DMFS, not shown), while the probeset that can detect both ERRβ2 and ERRβ-Δ10 shows a significant positive correlation with longer RFS (hazard ratio 0.56, log-rank p = 0.00092) and DMFS (hazard ratio 0.48, log-rank p = 0.035). No probesets that can detect ERRβsf (alone or in combination with others) are available on this Affymetrix platform. Garattini et al.'s recent analysis of nuclear receptor superfamily expression in The Cancer Genome Atlas (TCGA) breast cancer RNAseq data shows that total ESRRB expression (referred to in the manuscript as NR3B2) is significantly reduced in breast tumors vs. normal breast tissue, with lowest expression in the Luminal B and Basal-Like molecular subtypes [90]. While preliminary, these data suggest that ERRβ2 may be a good prognostic factor in TNBC, and are consistent with the findings we present here that implicate the activated ERRβ2 splice variant as a potent inhibitor of mitotic progression in breast cancer cells, including triple negative models.

## MATERIALS AND METHODS

### Cell culture

MCF10A non-transformed mammary epithelial cells, and MCF7 and MDA-MB-231 breast cancer cells, were obtained from the Lombardi Comprehensive Cancer Center (LCCC) Tissue Culture Shared Resource. HCC1806 and MDA-MB-468 breast cancer cells were purchased from ATCC (Manassas, VA). Cells routinely tested negative for *Mycoplasma spp*., and were fingerprinted by the Tissue Culture Shared Resource to verify their authenticity using the 9 standard STR loci and Y chromosome-specific amelogenin. All cells were maintained in a humidified incubator with 95% air: 5% carbon dioxide. MCF7 and MDA-MB-231 cells were grown in improved minimal essential media (IMEM; Life Technologies, Grand Island, NY) supplemented with 5% heat-inactivated fetal bovine serum (FBS, purchased from the LCCC Tissue Culture Shared Resource). HCC1806 and MDA-MB-468 cells were cultured in IMEM with 10% FBS. MCF10A cells were grown in a 1:1 mixture of Ham's F12: Dulbecco's modified essential media (DMEM) supplemented with 20 ng/ml epidermal growth factor (EGF), 10 μg/ml insulin, 0.5 μg/ml hydrocortisone, 100 ng/ml cholera toxin, and 5% horse serum (all purchased from the LCCC Tissue Culture Shared Resource).

### General reagents

Geneticin (G418, Life Technologies) was used at a final concentration of 1.2 mg/ml for MCF7 cells stably expressing fluorescent histone H2B. ERRβ agonists DY131 and GSK4716, and the p38 inhibitor SB203580 (Tocris Bioscience, Ellisville, MO), were dissolved in dimethyl sulfoxide (DMSO; Sigma Aldrich, St. Louis, MO) at a concentration of 10 mM, stored at −20°C, and used at the indicated concentrations. The microtubule inhibitor nocodazole (Sigma Aldrich), Smoothened inhibitors vismodegib and cyclopamine (kind gifts from Dr. Insoo Bae), paclitaxel (generously provided by Dr. Robert Clarke), flavopiridol (kind gift from Dr. Christopher Albanese), and the ATM inhibitor KU-55933 (generously provided by Dr. Gil Palchik) were also prepared as concentrated stocks in DMSO, stored at −20°C or 4°C (nocodazole), and used at the indicated concentrations. Doxorubicin (kind gift from Dr. Robert Clarke), mitoxantrone (generously provided by Dr. Rabindra Roy), and arsenic trioxide (ATO, [54]) were prepared as concentrated stocks in molecular biology-grade water, stored at −20°C, and used at the indicated concentrations. Human ERRγ purified protein (transcript variant 2), was purchased from Origene (Rockville, MD).

### Plasmids and transfection

The ERE-luciferase, ERRE-luciferase (Addgene #37851), 8xGLI1-luciferase, and pRL-SV40-Renilla promoter-reporter constructs have been previously described [42, 43, 54, 91]. The pSG5 empty vector, ERRγ, ERRβsf (Addgene #52188), ERRβ2 (Addgene #52186), and EGFP-GLI1 expression constructs have also been published previously [27, 43, 54, 92]. FLAG-AIB1 was generously provided by Dr. Anna T. Riegel [36]. H2B-GFP was a gift from Dr. Geoff Wahl (Addgene plasmid # 11680) [67]. Plasmids were introduced to cells using either jetPRIME (Polyplus Transfection, Ilkirch, France) or Lipofectamine LTX (Life Technologies) according to manufacturer's instructions. After 4-5 h, transfection complexes were removed and fresh media were added as appropriate.

### Dual-luciferase promoter-reporter assays

Cells were seeded into 24-well plastic tissue culture dishes (35,000 per well for MDA-MB-231; 50,000-75,000 per well for MCF7) on day 0, transfected on day 1 with a total of 500 ng DNA/well (100-200 ng receptor, EGFP-GLI1, or AIB1 expression plasmid; 195-240 ng luciferase reporter plasmid; 5-10 ng Renilla control), treated 4-5 h post-transfection for 18-24 h with the indicated compounds, and harvested on day 2 for dual-luciferase assay as described in [27]. Luciferase activity was normalized to Renilla activity. Each experiment was performed in triplicate.

### Cell proliferation assays

Cells were seeded into 3, 96-well plastic tissue culture dishes per line at 1,000 cells per well on day 0. On day 1, each plate was treated with the indicated concentrations of DY131. Plates were re-dosed every 3 days and stained on days 3 or 4, 7 or 8, and 10 or 11. To stain, one plate per line was rinsed once with 1X Phosphate-Buffered Saline (PBS) and incubated with a solution of 0.5% w/v crystal violet (Sigma Aldrich) dissolved in 25% methanol: 75% water at 4°C for 10 minutes. Excess stain was removed and each plate was washed 5-6 times with deionized H2O and allowed to air dry completely. Stained cells were rehydrated in a 0.1M sodium citrate buffer dissolved in 50% ethanol: 50% water, then read on a plate reader at an absorbance of 550nm. Each experimental condition was performed in six replicate wells.

### Colony formation assays

On day 0, 150 (MDA-MB-231) or 200 (MCF7) cells were seeded per well in 4 wells of a 12-well plastic tissue culture dish. The following day, indicated concentrations of DY131 were added for 18-24 h. On day 2, drug-containing media were removed, wells were washed with 1X PBS, and fresh media (no drug) was added to the wells. Cells were cultured in the absence of drug for an additional 13 d, changing media twice, before staining with crystal violet solution as above.

### Total cell lysis and subcellular fractionation

Preparation of whole cell extracts in radioimmunoprecipitation assay (RIPA) buffer supplemented with protease and phosphatase inhibitors from cells seeded in 6-well plastic tissue culture dishes and (as appropriate) transfected or treated was carried out as in [27]. Samples to be probed for expression or post-translational modification of histones were gently sonicated prior to protein quantification (30% output, 3 seconds on/3 seconds off for a total of 3 times). Subcellular fractionation was carried out using a modification of the REAP method [93]. Cells were washed three times with cold PBS, aspirating in between washes. Then 1 mL of cold PBS was added to the 10 cm dish, and cells were scraped into an Eppendorf tube and centrifuged at 4°C in a microcentrifuge for 5 minutes at 5000 x g. On ice, 1 mL of 0.4% Nonidet P 40 substitute was added to resuspend the cell pellet. 300 μL of the total cell lysate was immediately taken from the resuspension and and centrifuged at 10,000 x g for 2 minutes at 4°C. The supernatant was added to 100 μL 4X loading buffer and boiled at 99°C for 8 minutes. The remainder of the lysate was incubated on ice for 3 minutes, then centrifuged at 4°C for 5 minutes at 5000 x g. Another 300 μL of the supernatant was collected as the cytosolic fraction and added to 100 μL of 4X loading buffer and then boiled at 99°C for 8 minutes. The nuclear pellet was then resuspended in 1 mL of 0.4% Nonidet P 40 substitute and centrifuged at 4°C for 2 minutes at 10,000 x g. The supernatant was discarded and the pellet was resuspended in 80 μL of 4X loading buffer and boiled at 99°C for 10 minutes.

### Western blotting and antibodies

Following polyacrylamide gel electrophoresis and protein transfer, nitrocellulose membranes were blocked in 5% nonfat dry milk*** dissolved in Tris-Buffered Saline with Tween-20 (TBST), then probed overnight at 4°C with the following primary antibodies in TBST: ERRbeta #PP-H6707-00 (cl.07) 1:250 - 1:500 and #PP-H6705-00 (cl.05) 1:500 – 1:750 (R&D Systems, Minneapolis, MN); PARP #9542 1:1000, phospho Ser139 (γ) histone H2A. X #9718 1:1000, ***total histone H2A. X #2595 1:1000, phospho Ser1981 ATM #5883 1:500, phospho Ser428 ATR #2853 1:500, phospho Thr68 Chk2 #2197 1:500, phospho Ser345 Chk1 #2348 1:500, phospho Ser10 histone H3 #3377 1:1000, total histone H3 #9715 1:1000, phospho Thr180/Tyr182 p38 MAPK #9216 1:250, p38 #9212 1:500, vinculin #13901 1:1000 (Cell Signaling, Danvers, MA). All membranes were re-probed with β-actin (Sigma #A5316 1:5000 – 1:10,000) as a loading control for ≥1 h at room temperature or overnight at 4°C. Horseradish peroxidase enzyme-conjugated anti-mouse or anti-rabbit whole immunoglobulin (IgG) secondary antibodies (GE #NXA931 and #NA934V, respectively, Buckinghamshire, U.K.) were used at 1:5000 for ≥1 h at room temperature, followed by enhanced chemiluminescence (ECL, Denville Scientific, Holliston, MA) as in [27]. ***Membranes to be probed for total histone H2A. X were blocked in 5% horse serum in TBST rather than milk.

### Cell cycle analyses

On day 0, cells were seeded at 100,000 – 150,000 cells per well in 6-well plastic tissue culture dishes one day prior to treatment with the indicated concentrations of drug. For experiments with the p38 inhibitor SB203580, cells were pretreated with the compound for 1 h before the addition of DY131. The following day (day 2), cells were collected, ethanol-fixed, stained with propidium iodide, and analyzed for cell subG1 (fragmented) DNA content and cell cycle profile as in [27].

### Apoptosis/necrosis assays

Cells were seeded and drug-treated as described above for cell cycle analyses. 12 h (MDA-MB-231), 16 h (HCC1806), or 24 h (MCF10A, MCF7, MDA-MB-468) post-treatment, cells were collected and stained with fluorescein isothiocyanate (FITC)-conjugated Annexin V and propidium iodide, and analyzed by flow cytometry as in [27].

### BIAcore DNA binding assays

DNA binding studies were performed in a Biacore T100 system (BIAcore, Uppsala, Sweden) as published previously [94, 95]. Briefly, the affinity of DY131, GSK4716, and mitoxantrone for DNA was tested using a 50-mer oligonucleotide:

5′-TCGAGGATCCTGAGCTCGAGTCGACGATCGCGAATTCTGCGGATCCAAGC-3′

The oligonucleotide was biotinylated and immobilized on streptavidin-coated C1 BIAcore chips as single-stranded DNA or in duplex with the reverse complement oligonucleotide as double-stranded DNA. Relative Unit (RU) values were recorded with three, 60s injections of each compound (15 μM) in a binding buffer containing 10 mM HEPES-KOH, pH 7.6, 90 mM KCl, and 0.05% surfactant P20 (BIAcore).

### Live-cell confocal microscopy

MCF7-GFP-H2B cells were seeded into 6-well glass-bottom tissue culture dishes at 150,000 cells per well one day before being synchronized by exposure to 100 nM nocodazole for 18-22 h. The following day, plates were brought to the LCCC Microscopy & Imaging Shared Resource's Nikon Eclipse TE-300 Inverted Spinning Disk Confocal Microscope System with heated and humidified environmental chamber and CO_2_ control. Cells were allowed to equilibrate in the chamber for 30 – 45 m, then five fields per condition with ≥1 cells per field with condensed chromatin were selected for imaging. Nocodazole-containing media were carefully removed, cells were washed twice with warm PBS, then immediately exposed to media containing DMSO vehicle control, 5 μM DY131, 10 μM DY131, 10 μM flavopiridol, or 250 nM paclitaxel. Images were acquired using a 20X/0.5 N.A. Plan Fluor Nikon objective. The microscope is coupled with a Perkin Elmer VoX core unit for spinning disk confocal imaging and image capture was via an EM-CCD cooled 1K X 1K CCD camera and Volocity Ver. 6.3 Acquisition software. An AOTF-controlled 488 nm laser diode was used for multi-well, live imaging of nuclear GFP-H2B. 10 image z-stacks at 1 μm spacing were obtained for each time point for multiple fields of view via a Prior Pro Scan motorized x, y, z stage.

### Fixed-cell confocal microscopy

For spindle polarity studies, cells were seeded onto 18 mm glass coverslips placed in 12-well plastic tissue culture dishes at 70,000 cells per well two days prior to staining, allowing for 24 h treatment with DY131 or DMSO control prior to fixation. For exogenous ERRβ subcellular localization studies, 200,000 - 300,000 cells per well were seeded two days prior to staining, allowing for 18-20 h transfection with ERRβsf or ERRβ2 cDNAs. For endogenous ERRβ/γ-tubulin co-localization studies, 85,000 cells per well were seeded one day prior to staining. Media were removed and cells were fixed and permeabilized in 3.2% paraformaldehyde with 0.2% Triton X-100 in PBS for 5 minutes at room temperature. For endogenous ERRβ/γ-tubulin co-localization studies, coverslips were further permeabilized in cold absolute methanol at −20°C for 10 minutes. Following three washes with PBS, coverslips were inverted onto one drop (~100 μl) of primary antibody in antibody diluent (0.1% gelatin with 10% normal donkey serum in PBS) on strips of parafilm and incubated as shown in the table found in the **Supplementary Materials**. Coverslips were stained first for ERRβsf or ERRβ2, where appropriate, then for γ-tubulin at room temperature. Antibody controls were incubated in antibody diluent only. After carefully lifting each coverslip and washing three times with PBS, coverslips were inverted onto one drop of the appropriate secondary antibody, DAPI dihydrochloride, and (where appropriate) ActiStain-488-phalloidin (Cytoskeleton, Denver, CO) in antibody diluent, then incubated in the dark at room temperature as shown in the **Supplementary Materials**. Coverslips were again washed with PBS, then gently dipped twice into molecular biology-grade water before inversion onto one drop of FLUOROGEL (Electron Microscopy Sciences, Hatfield, PA) before being allowed to air-dry in the dark for at least 10 minutes. Slides were stored at 4°C until image collection on the LCCC Microscopy & Imaging Shared Resource's Zeiss LSM510/META/NLO multi-photon microscope using the settings shown in the table found in the **Supplementary Materials**.

### Image and statistical analyses

Photoshop CreativeSuite 5.1 was used to assemble figures, FIJI (http://fiji.sc/Fiji) was used to perform densitometry on scanned Western blots, and Volocity 3D Image Analysis Software (PerkinElmer, Waltham, MA) was used to analyze confocal microscopy data (see Supplementary Materials). All statistical analyses, except those in Supplementary Figure S4, were performed in Prism 6.0 (Graphpad, San Diego, CA), and are specified in the figure legends. All data are presented as the mean ± standard deviation (S.D.), with the exception of Figure 9C and 9D, where data are depicted as ‘parts of the whole’ plots. Statistical significance is defined as a *P* value of ≤0.05. *p≤0.05, **p≤0.01, ***p≤0.001, ****p≤0.0001.

## SUPPLEMENTARY METHODS FIGURES AND MOVIES












