# Supplementary material for: Antimitotic activity of DY131 and the estrogen-related receptor beta 2 (ERRβ2) splice variant in breast cancer
Source: Oncotarget. 2016 May 30;7(30):47201–20. doi: 10.18632/oncotarget.9719 (PMC5216935; doi:10.18632/oncotarget.9719)
Supplement: Supplementary file 1 [file oncotarget-07-47201-s001.pdf]

Antimitotic activity of DY131 and the estrogen-related receptor beta 2 (ERRβ2) splice variant in breast cancer

SUPPLEMENTARY METHODS

Antibody table for immunofluorescence studies

Antibody, species	Vendor	Dilution, time
ERRβ2, cl. 07, mouse	Perseus Proteomics	1:150, 1 hr.
ERRβsf, cl .05, mouse	Perseus Proteomics	1:150, 1 hr.
γ-tubulin, rabbit	Novus, #NB120-11318	1:500, 25 min.
AlexaFluor-594, anti-rabbit or anti-mouse	Life Technologies	1:200, 20 min.
AlexaFluor-488, anti-rabbit or anti-mouse	Life Technologies	1:500, 20 min.
ActiStain-488-phalloidin	Cytoskeleton	1:300, 20 min.
DAPI dihydrochloride	Life Technologies	1:500, 20 min.

Zeiss LSM510/META/NLO Multi-photon microscope settings

Fluorophore	Settings
AlexaFluor-594 secondary	20% intensity, dichroic mirror 545, long pass filter 560
AlexaFluor-488 secondary	15% intensity, dichroic mirror 490, band pass filter 525/50
DAPI dihydrochloride	0.4% intensity, 770 nm excitation, band pass filter 460/50

Determination of time to anaphase following release of nocodazole block

Volocity Visualization was used to generate 3D Opacity and Extended View renderings of nuclei from time-lapse recordings. Time to anaphase after nocodazole release was determined by inspection of cells that appeared to be in prophase at the outset of imaging. Time-lapse images were taken every 6.83 minutes and time to anaphase was determined as the first frame where two (or more) separate daughter cells were observed. Typically, the daughter cells also had reduced GFP intensity compared with the metaphase plate. Mitosis of cells that were not in prophase in the first time point after nocodazole release but which later entered prophase and underwent division was also analyzed. In this case, prophase, metaphase, and anaphase were observed as the onset of chromosome condensation with increased GFP average intensity (prophase), maximum GFP intensity and minimum volume (metaphase plate in varying 3D orientations), and the appearance of separate daughter cells with reduced average GFP intensity (anaphase).

Visual observations were supplemented with 3D analysis of total intensity and volume of nuclear objects using Volocity software version 6.3 (Perkin Elmer, Waltham, MA). Nuclei identified by GFP-H2B were selected in each image frame first by cropping an image volume containing only a single cell. Next Volocity was used to track the GFP-H2B labeled nucleus from frame to frame, recording the total intensity and volume at each time point. To start the analysis, thresholding was adjusted together with min and max volumes so that parent and daughter cells were optimally identified in each timepoint. The same routine was subsequently applied to each cell analyzed. Mis-tracking was sometimes identified after cell division particularly when the daughter cells came in contact with each other or when the daughter cells failed to separate completely during the time course, frequently in the case of 10 μm DY131 treated cells. See figure below: in A, the red arrow indicates the assignment of anaphase at timepoint 37 and the images are of timepoints 30-43; in B, the graphs show total intensity and volume with time. Only a segment of the time course is shown for clarity, timepoints 30-43 are indicated by the labeled bar; red arrows indicate timepoint 37, anaphase).

A.

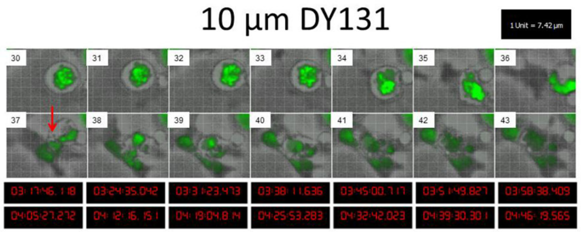

B.

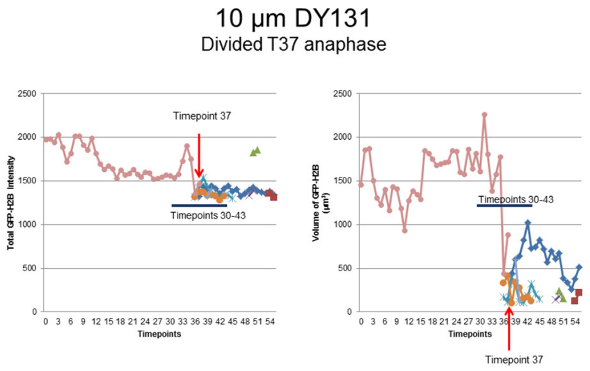

GFP_XY point 21 (well B2) (cropped2)

REFERENCES

1. Costes SV, Daelemans D, Cho EH, Dobbin Z, Pavlakis G, Lockett S: Automatic and quantitative measurement of protein-protein colocalization in live cells. Biophys J 2004;86:3993-4003.
2. Györffy B, Lanczky A, Eklund AC, Denkert C, Budczies J, Li Q, Szallasi Z: An online survival analysis tool to rapidly assess the effect of 22,277 genes on breast cancer prognosis using microarray data of 1,809 patients. Breast Cancer Res Treat 2010;123:725-731.
3. Goldhirsch A, Winer EP, Coates AS, Gelber RD, Piccart-Gebhart M, Thürlimann B, Senn HJ, members P: Personalizing the treatment of women with early breast cancer: Highlights of the st gallen international expert consensus on the primary therapy of early breast cancer 2013. Ann Oncol 2013;24:2206-2223.

SUPPLEMENTARY FIGURES AND MOVIES

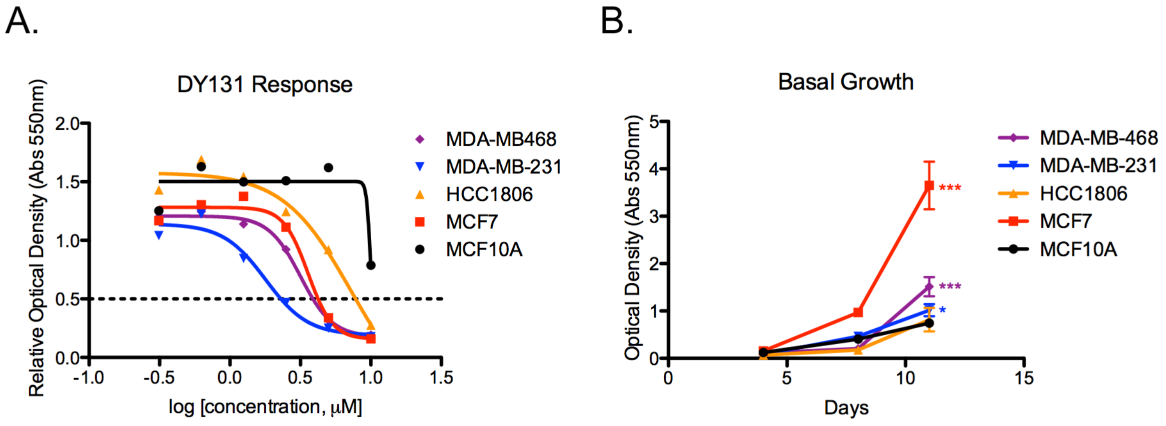

Supplementary Figure S1: DY131 response and basal growth in breast cancer and immortalized breast epithelial cell lines. A, Crystal violet staining of MCF10A, MCF7, HCC1806, MDA-MB-231, and MDA-MB-468 in the presence or absence of the indicated concentrations of DY131 on Day 7/8 from data presented in Figure 2A. Data for each cell line were normalized to the appropriate DMSO control, followed by curve fitting to sigmoidal dose-response (variable slope) parameters in GraphPad Prism 6.0. Dashed line denotes approximate IC_{50}. B, Crystal violet staining of MCF10A, MCF7, HCC1806, MDA-MB-231, and MDA-MB-468 grown in the absence of DY131 (DMSO control curves for each cell line from data presented in Figure 2A). N = 6 for a representative assay performed in sextuplicate, two-way ANOVA with Bonferroni post-tests for each cell line vs. MCF10A.

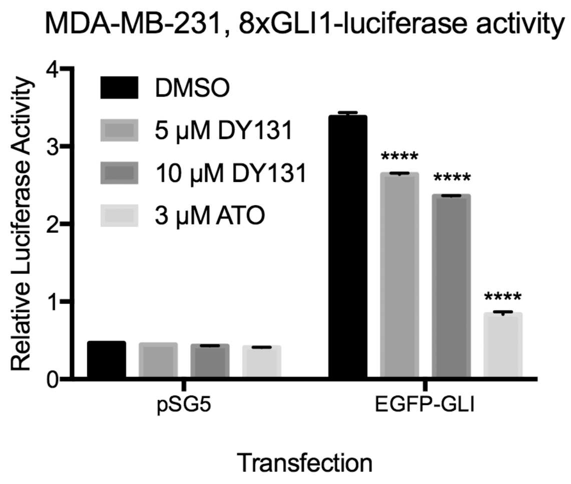

Supplementary Figure S2: DY131 modestly inhibits GLI-luciferase activity. MDA-MB-231 cells transiently co-transfected with the indicated promoter-reporter luciferase constructs and EGFP-GLI1 cDNA, then treated with either DY131 (DY), GLI inhibitor arsenic trioxide (ATO), or DMSO control (18-20 h) as shown. N = 3 for a representative assay shown in triplicate, two-way ANOVA with Bonferroni post-tests for each treatment vs. DMSO.

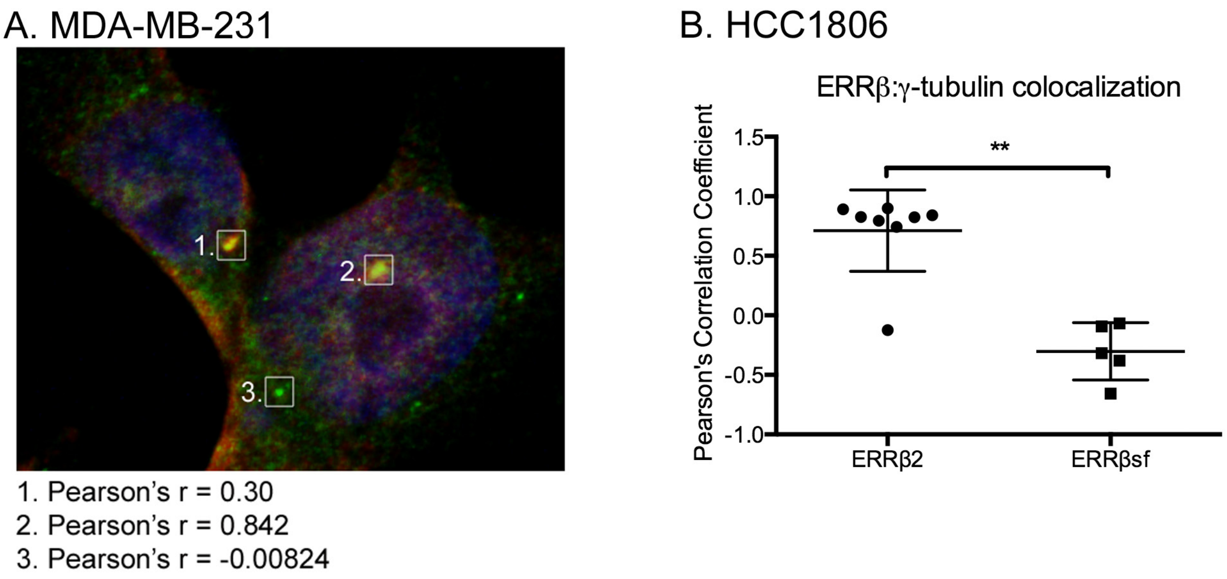

Supplementary Figure S3: Correlations for signal intensity of ERRβ2 and γ-tubulin colocalization. A, Enlargement of MDA-MB-231 cells immunostained with ERRβ2 (mouse monoclonal cl.07 antibody, green), γ-tubulin (rabbit polyclonal antibody, red), and DNA (DAPI). The images were imported into Volocity and presented here using Volocity Visualization module, 3D Opacity mode. Pearson correlation coefficients for red/green signal overlap (shown for three discrete spots contained within the indicated ROIs) were calculated by Volocity using the Quantitation module. The colocalization analysis was conducted using automatic thresholding published by Costes et al [1]. Values of 1 indicate the red and green pixel intensities are perfectly correlated, 0 indicates no correlation, and values closer to -1 indicate inverse correlations. Spot 3 was selected as a negative control. B, Quantification of red/green signal overlap for ERRβ2 or ERRβsf (red) with γ-tubulin (green) in HCC1806 cells. N = 5 – 8, Mann-Whitney Test.

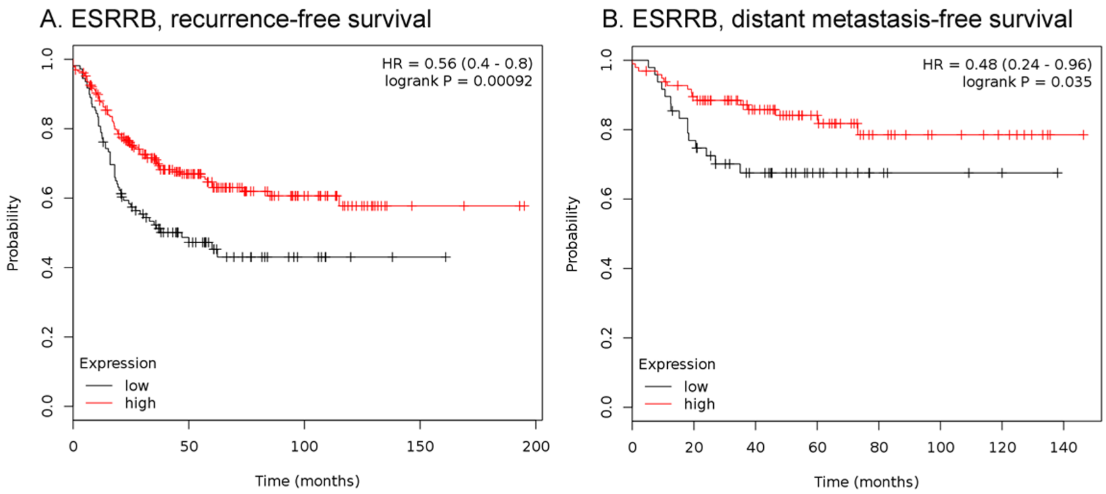

Supplementary Figure S4: Association of ERRβ expression with survival in basal-like breast cancer. KMPlot [2] was used to query ESRRB expression in breast tumors categorized as basal (ESR1-/HER2-) according to the 2013 St. Gallen Consensus [3], with the endpoint of improved recurrence-free (A) and distant metastasis-free survival (B). Probeset ID 223858_at on the Affymetrix U133 Plus 2.0 platform detects both ERRβ2 and ERRβ-Δ10. No probesets on this platform can detect ERRβsf.

SUPPLEMENTARY MOVIES

MCF7 cells stably transfected with GFP-H2B were synchronized by exposure to 100 nM nocodazole overnight (18-22h), then released into media containing DMSO control or the indicated compound.

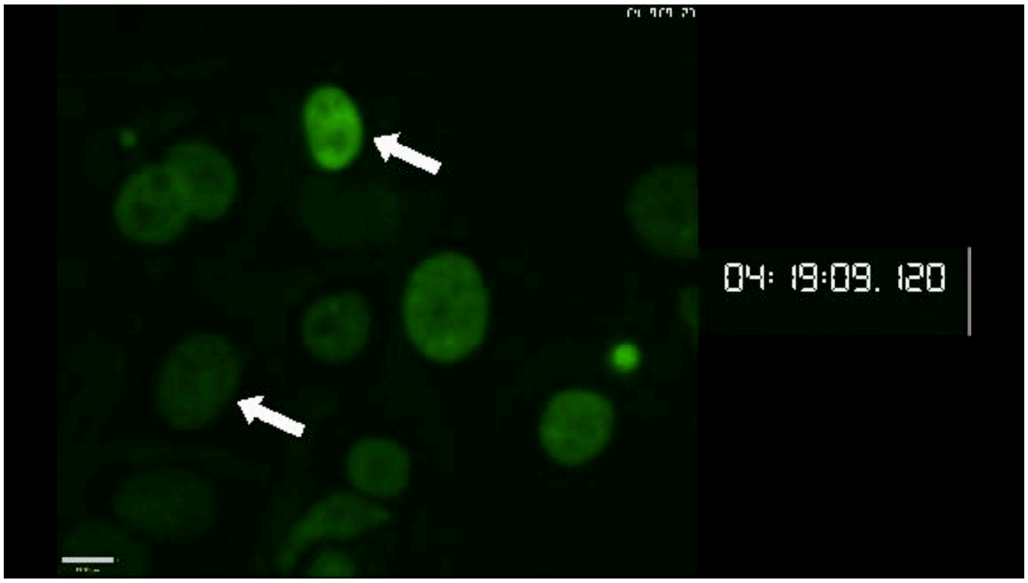

Supplementary Movie S1: Time-lapse of cells released into 10 µM flavopiridol.

See Supplementary Movie File 1

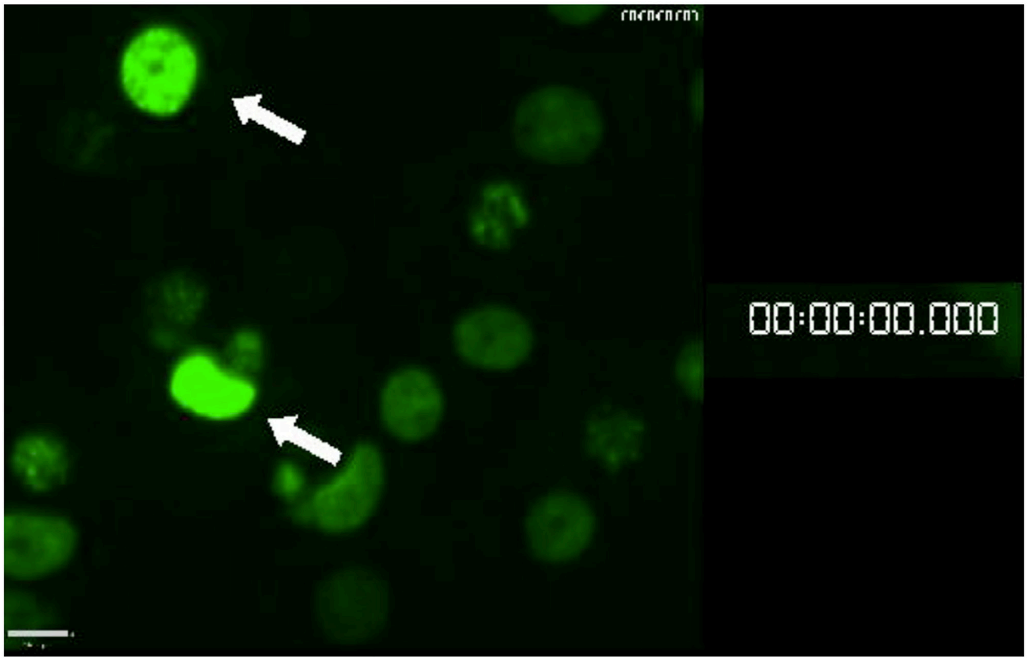

Supplementary Movie S2: Time-lapse of cells released into 250 nM paclitaxel.

See Supplementary Movie File 2

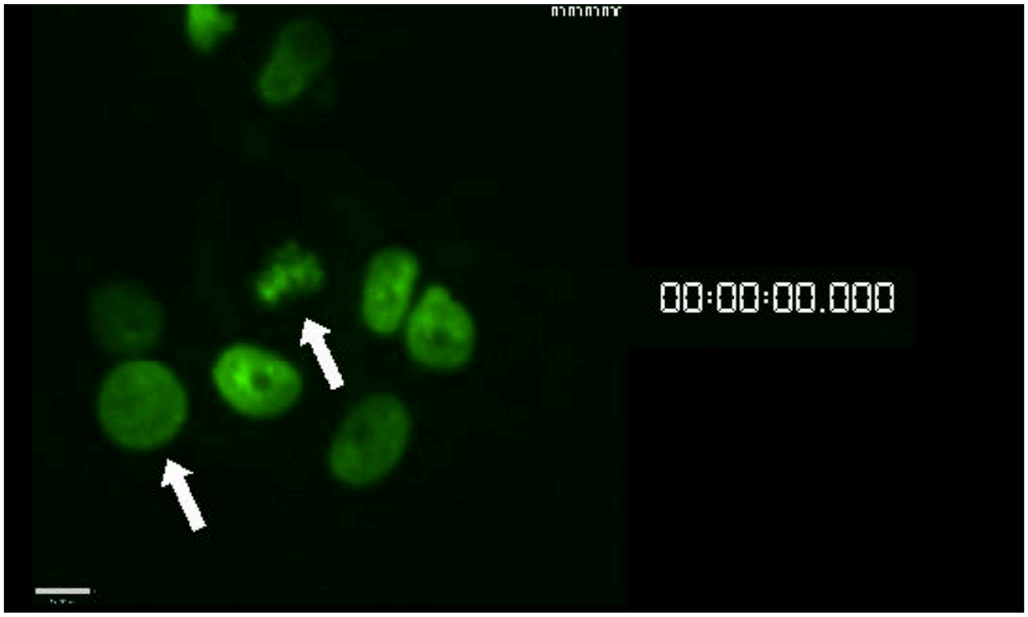

Supplementary Movie S3: Time-lapse of cells released into DMSO.

See Supplementary Movie File 3

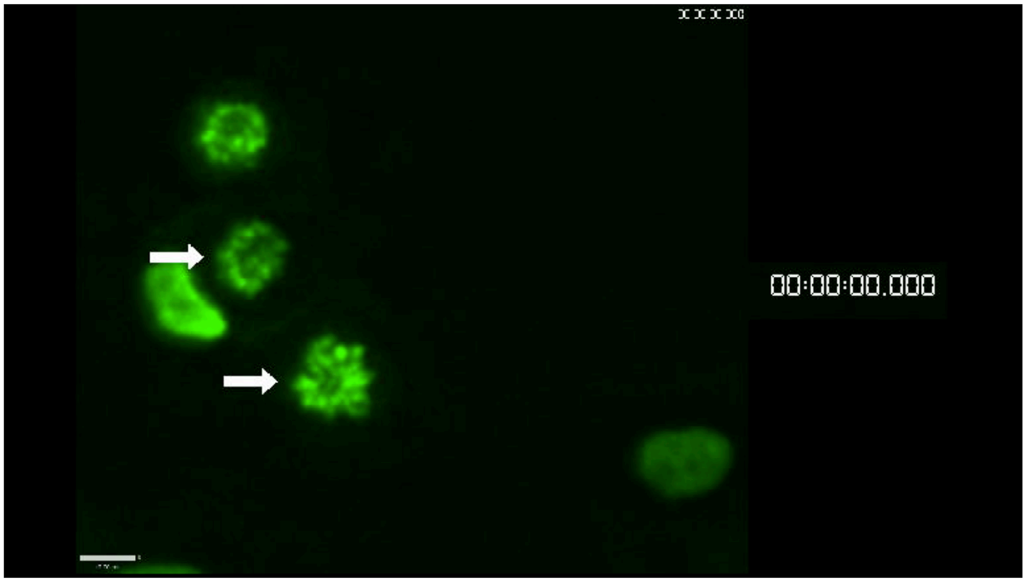

Supplementary Movie S4: Time-lapse of cells released into 5 µM DY131.

See Supplementary Movie File 4

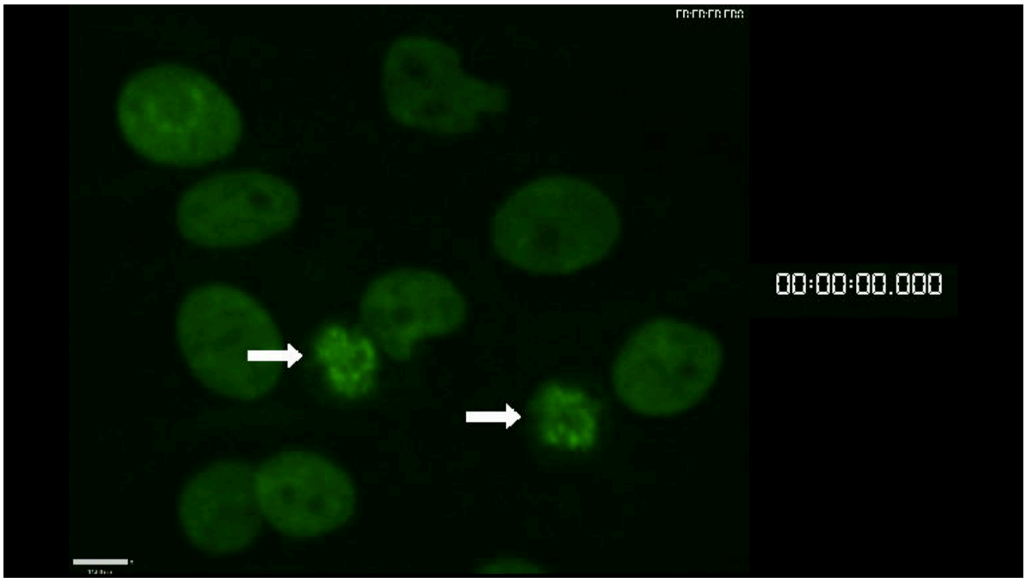

Supplementary Movie S5: Time-lapse of cells released into 10 µM DY131.

See Supplementary Movie File 5
